# Supplementary material for: Utilization of insecticide-treated bed nets among pregnant women in Myanmar–analysis of the 2015–2016 Demographic and Health Survey
Source: PLoS One. 2022 Mar 10;17(3):e0265262. doi: 10.1371/journal.pone.0265262 (PMC8912190; doi:10.1371/journal.pone.0265262)
Supplement: Attachment — Submitted filename: Comments to the authors ITN.pdf [file pone.0265262.s002.pdf]

General comments

The overall structure of the manuscript is good and written based on the commonly agreed structure. The discussion and conclusion section needs major revision based on the objective and the result of the study.

Specific comments

1. **Title**: it can be modified "utilization of insecticide treated bed nets among pregnant women in Myanmar: analysis of 2015-2016 demographic and health survey". Because no need of mentioning ---to prevent malaria transmission… as it is known.
2. **Abstract**

2.1 Background- it lacks information why the authors did the analysis.

2.2 Result- The description of the use of bed net is not clear. It stated that 16% slept without bed net and 18.4 slept under ITN. What about others…16% +18.4%=34.4%, 65.6% are they users or not. Make it clear for the reader. In the main document, it is clear.

Mentioning the result of bivariate analysis in the abstract is not important.

2.3 Conclusion- the recommendation sentence is not based on the significant variable. Mass media exposure do not show a significant association with utilization.

Keywords: it is good if arranged alphabetically

3. **Introduction**
    - Reference number one is outdated you can update and use the 2021 malaria report which indicates 241 million cases of malaria. (https://www.who.int/news-room/fact-sheets/detail/malaria)

- Based on line 51 and 52 sentence, malaria is not a problem of pregnant women. How do the authors explain the public health importance of this research for Myanmar?
- It is good if the authors indicate the interventions by different stakeholders to increase ownership and utilization of ITN among pregnant women.

4. **Method**

- It is good if line number 126 to 138 presented under the introduction section, which shows the intervention by different stakeholders.

5. **Result**

- Table 1- the bracket under percentage column for all percent is not necessary as it is written in a separate column. In the table the frequency added to 99.9% not 100%, check it.
- Line 196 seems discussion, not description of the result. It can be stated in the discussion section.
- Line 207-215 should be under bed net utilization, not the factors associated with bed net utilization.

6. **Discussion**

- The discussion should be guided by the objective of the study. However, most of the parts of the discussion is based on the general understanding not based on the result of the study. Only paragraph 2 and 3 focused discussion based on the objective.
- It lacks the limitation of the study. Many variables may be missed because it is a secondary data such as knowledge, attitude, risk perception etc.

7. **Conclusion**

- The recommendation part (line 310-318) is a general recommendation not based on the result of the study. Mass media and other intervention suggested is not the significant variables, which is associated with utilization of ITN in this study.
