# Supplementary material for: Utilization of insecticide-treated bed nets among pregnant women in Myanmar–analysis of the 2015–2016 Demographic and Health Survey
Source: PLoS One. 2022 Mar 10;17(3):e0265262. doi: 10.1371/journal.pone.0265262 (PMC8912190; doi:10.1371/journal.pone.0265262)
Supplement: Attachment — Submitted filename: Reviewers comment for PONE-D-22-00065.docx [file pone.0265262.s001.docx]

1.	Is the manuscript technically sound, and do the data support the conclusions?

The manuscript must describe a technically sound piece of scientific research with data that supports the conclusions. Experiments must have been conducted rigorously, with appropriate controls, replication, and sample sizes. The conclusions must be drawn appropriately based on the data presented.

Reviewer: Yes

2. Has the statistical analysis been performed appropriately and rigorously?

Reviewer: No

3. Have the authors made all data underlying the findings in their manuscript fully available?

The PLOS Data policy requires authors to make all data underlying the findings described in their manuscript fully available without restriction, with rare exception (please refer to the Data Availability Statement in the manuscript PDF file). The data should be provided as part of the manuscript or its supporting information, or deposited to a public repository. For example, in addition to summary statistics, the data points behind means, medians and variance measures should be available. If there are restrictions on publicly sharing data—e.g., participant privacy or use of data from a third party—those must be specified.

Reviewer: Yes

4. Is the manuscript presented in an intelligible fashion and written in standard English?

PLOS ONE does not copyedit accepted manuscripts, so the language in submitted articles must be clear, correct, and unambiguous. Any typographical or grammatical errors should be corrected at revision, so please note any specific errors here.

Reviewer: Yes

5. Review Comments to the Author

Thank you very much for this opportunity to revise the manuscript titled "Utilization of insecticide-treated bed nets to prevent malaria transmission among pregnant women in Myanmar – A secondary data analysis of the 2015-2016 nationwide Demographic Health Survey" that was submitted to PLOS ONE. The study is original research that utilized secondary data from 2015-2016 nationwide Demographic Health Survey-Myanmar. The study revealed very low rate of insecticide-treated bed nets usage among pregnant women. Insecticide-treated bed nets are the simplest way of preventing mosquito bites. Mosquito bites causes malaria. Malaria infection affects all persons in malaria endemic areas, however pregnant women and children under five are more vulnerable. In pregnancy, Malaria can cause anaemia, which lead to stillbirths and in some instance maternal morbidity and mortality. Due to the effectiveness of ITNs, most malaria endemic countries resort to free distributions to the population with special attention to the expectant mothers and postnatal mothers. However, in most countries the mere distribution of ITNs do not translate to utilisation. From above therefore, this study therefore is considered very important for policy directions. However, for this work to be published in Plos One, these are some comments for the authors in order to improve the manuscript:

**Abstract**

Methods under Abstract ought to be improved.

•	Indicate the version of STATA used for the analysis.

•	The study design in missing in this section.

•	At what point, is a variable said to be significant in the inferential statistics?

**Introduction**

The authors begin talking about Insecticides Treated bed Nets. However, in many European countries, the use of this method of malaria prevention is little known, so it could be interesting to add a sentence explaining what it consists of and what health benefits it has. This will help readers form countries in which malaria is not endemic to appreciate the importance of this study.

The authors may refer to a study in Ghana for the definition and benefits of ITNs … ***Utilization of Insecticides Treated Mosquito Nets (ITNs) Among Pregnant Women in Kassena-Nankana Municipality***… Kindly refer to the introduction and cite appropriately.

-Kindly read the following published work in Myanmar and update line 78 to 81…. ***Other studies in Thailand and Myanmar stated that types of occupation, gender, age, socioeconomic status, net ownership, and residing places ….***

Liu, H., Xu, J. W., Guo, X. R., Havumaki, J., Lin, Y. X., Yu, G. C., & Zhou, D. L. (2015). Coverage, use and maintenance of bed nets and related influence factors in Kachin Special Region II, northeastern Myanmar. Malaria journal, 14(1), 1-12.

Cheng, B., Htoo, S. N., Mhote, N. P. P., & Davison, C. M. (2021). Association between biological sex and insecticide-treated net use among household members in ethnic minority and internally displaced populations in eastern Myanmar. Plos one, 16(6), e0252896.

**Methods**

-Kindly specify if the study design is descriptive or analytical cross-sectional study

-Line 126 to 138 including the figure 1 should be moved to introduction.

- Kindly make a sub heading for dependent (outcome) and independent (explanatory) variables and provide vivid description to each.

- The criteria for inclusion into the logistics was not stated. This has the tendency affect the goodness of fit of the model.

See the following (concentrate on the data analysis only);

Mekonnen, B. D., & Wubneh, C. A. (2020). Prevalence and associated factors of contraceptive discontinuation among reproductive-age women in Ethiopia: using 2016 Nationwide Survey Data. *Reproductive Health*, *17*(1), 1-10.

Abubakari, A., Taabia, F. Z., & Ali, Z. (2019). Maternal determinants of low birth weight and neonatal asphyxia in the Upper West region of Ghana. *Midwifery*, *73*, 1-7.

Mutaru, A. M., Asumah, M. N., Ibrahim, M., Sumaila, I., Hallidu, M., Mbemah, J. M., ... & Zakaria, D. Y. (2021). Knowledge on Sexually Transmitted Infections (STIs) and sexual practices among Nursing Trainees in Yendi Municipality, Northern Region of Ghana. *European Journal of Health Sciences*, *6*(4), 33-47.

**Results**

-Table 1; kindly check the percentages of Wealth index, it is more than 100.0%. Kindly check the values at 1 decimal place again and make necessary changes.

-Line 194, the authors discovered that 96% of the pregnant women possessed bed nets. However, in figure 2 no such information is captured. kindly reconcile the descriptions and figure 2.

-For consistency in reporting of findings …I suggest the authors reports findings as … 96% (95% CI: 93.2, 97.7)

-line 195-198, it is indicated that… “*However, 16% (95% CI: 12.4, 20.2) of pregnant women slept without a bed net last night prior to the survey which was a bit higher than the general population (12%). Many of both groups preferred to sleep under the untreated nets. Only about one in five (18.4%, 95% CI: 14.7, 22.9) pregnant women slept under insecticide-treated bed nets”.*

-From above, my understanding is that about 84% and 88% of pregnant women and general population respectively slept in bed nets a night to the survey. Is that correct?

-If above question is correct, how then did we get only 18.4% of pregnant women reported to be using *insecticide-treated bed nets?*

*_How do we know that the other nets were not treated? In the introduction, I came across free distribution of bed nets aimed at meeting the 2030 targets by the country. It is the case that, majority of the 96% ownership of bed nets are not treated? Kindly explain and make it clear to the reader.*

-The authors further indicated that, only 18.4% slept in ITNs. this is problematic. Because the dependent (outcome) variable was not clearly defined, it is becoming very difficult to understand these findings.

-I would suggest that the methods be clarified to give true meaning to these findings.

-Bed nets and *insecticide-treated bed nets should not be used interchangeably. Insecticide-treated bed nets are bed nets, but not all bed nets are insecticide-treated bed nets.*

*-I also suggest definition of terms at the methods to give true meaning to the findings.*

*_Line 207 -215 is not very necessary. I suggest to the authors to expunged expunge it.*

*-Only one variable is significant at the adjusted model because the authors did not control confounding variables. I would suggest that, only significant variables or variables with a p value less than 0.2 at* bivariate analysis *level be considered in the adjusted model analysis. This should be clearly stated at the methods. You may refer to the materials provided at the methods sections.*

***Discussions***

*-The discussion is okay and up to date, However, if authors would not mind, I would suggest they add some studies from Africa to support the work. I am suggesting very recent studies for your perusal and update. Africa is another malaria endemic area and almost all the interventions outline in the study are being done in most Africa countries.*

Ngouakam, H., Fru-Cho, J., & Tientche, B. (2021). Awareness, Use, Care of Insecticide-treated Bed Nets among Pregnant Women in Buea (Buea) and Bonassama (Douala). *Health Science Journal*, *15*(2), 1-8.

Asumah, M. N., Akugri, F. A., Akanlu, P., Taapena, A., & Boateng, F. (2021). Utilization of insecticides treated mosquito bed nets among pregnant women in Kassena-Nankana East municipality in the upper east region of Ghana. *Public Health Toxicology*, *1*(2), 1-11. (**Read to see why rural folks tend to use ITNs more than the urban folks, and the wealth index too at the discussion section**)

Tassembedo, M., Coulibaly, S., & Ouedraogo, B. (2021). Factors associated with the use of insecticide-treated nets: analysis of the 2018 Burkina Faso Malaria Indicator Survey. *Malaria Journal*, *20*(1), 1-9.

-Limitation of this study should be mentioned at the end of the discussions.
