# Supplementary material for: Utilization of insecticide-treated bed nets among pregnant women in Myanmar–analysis of the 2015–2016 Demographic and Health Survey
Source: PLoS One. 2022 Mar 10;17(3):e0265262. doi: 10.1371/journal.pone.0265262 (PMC8912190; doi:10.1371/journal.pone.0265262)
Supplement: Attachment — Submitted filename: PLOS REVIEWER AA.dot [file pone.0265262.s003.dot]

**Evaluation**

The authors focused on the ownership and utilisation insecticide treated nets among pregnant women in Myanmar. Women constitute with children, a major risk group and known to be vulnerable to the disease. Despite great achievement in malaria control during the past decade, due to various interventions, the disease remains a huge public concern in developing countries. This is due partly to low utilisation of ITNs and poor attitude to preventive measures. Therefore, this paper presented aimed at to understand factors associated with the non-utilization of ITNs among pregnant women in Myanmar. The manuscript revealed a relatively poor ITNs utilization rate pregnant woman in Myanmar which could jeopardise the attainment of the malaria elimination goal by 2030. Residing in plains and hilly areas were preditors of non-utilisation of ITNs among pregnant women in Myanmar. With regards to weakness, minor English revision may be useful. The manuscript was well written following PLOS ONE recommended outline. The manuscript represents a high level of scientific work and appear to be an interested topic for scientists works in the areas of ITNs universal coverage.

**The Title**: Utilization of insecticide-treated bed nets to prevent malaria transmission among pregnant women in Myanmar – A secondary data analysis of the 2015-2016 nationwide Demographic Health Survey. **Proposed Title**: Utilization of insecticide-treated bed nets among pregnant women in Myanmar – a secondary data analysis of the 2015-2016 nationwide demographic health survey.

**17- Abstract:**

**18- Background**: Myanmar is approaching to achieve the malaria elimination goal by 2030 (Please the authors should remove this sentence)

**29-Results:** Among them, 16% slept without a bed net last night prior to the survey and only about one in five (18.4%) slept under ITNs. Among them, 16% slept without a bed net the night prior to the survey while only about one in five (18.4%) did.

32-33-34: Please when the results of the adjusted analysis are given, include the p-value. example: Women who were residing in the delta and lowland region [adjusted Odd Ratio (aOR)=7.97, 95% Confidence Interval (CI): 3.73, 17.05], p-value, plains (aOR=7.16, 95%CI: 3.11, 16.46, p-value)

**37- Conclusions**: The authors stated that "To enhance the utilization of ITNs, targeted health education using mass media should be implemented". Can the authors ascertained that in Myanmar do this intervention is yet to be implemented in Myanmar?

**44-INTRODUCTION**

52-53. In Myanmar, 56,640 malaria cases and 14 malaria-related deaths were reported in 2019 and of them, none were reported as pregnant women (The authors should kindly rephrase this sentence).

74-75 Thus, it needs immediate attention to strengthen the use of ITNs especially in rural areas.

(Thus, there is a need for immediate attention to strengthen the use of ITNs especially in rural areas).

**97-Methods**: Well written and clearly stated

**179- RESULTS**

184-185. Many respondents 185 (59.3%) occupied 4-7 household members (Kindly rephrase the sentence).

194 Most of the pregnant women (96%, 95% CI: 93.2, 97.7) Please kindly include the p-value when reporting the Odd ratio.

195-196-However, 16% (95% CI: 12.4, 20.2) of pregnant women slept without a bed net last night prior to the survey which was a bit higher than the general population (Please the authors should kindly represent the p-value. This is a result section and the authors should not consider any comparison with the general population

197-Many of both groups preferred to sleep under the untreated nets (Please the authors should kindly rephrase this sentence).

208 nets (ITNs) last night before the survey. The night before the survey

211-212-213. Moreover, the ones who had the lowest wealth index (91.2%) and possessed 4 to 7 family members in their households (82.7%) did not utilize ITNs last night before the survey. (Moreover, those who had the lowest wealth index (91.2%) and possessed 4 to 7 family members in their households (82.7%) did not utilize ITNs the night before the survey).

215 Last, the majority (85.2%) were exposed to mass media at least once a week. (Please removed the word last at the beginning of the sentence).

216- In bivariate analysis (In the bivariate analysis).

**209. Discussion**

245-246. In this study, although most of the pregnant women possessed a kind of bed nets (The authors should clarify the kind of bed nets).

245- 246. In this study, although most of the pregnant women possessed a kind of bed nets, some did not sleep under bed nets while only a few slept under ITNs. (Authors should reconsider this sentence, there is a repetition of the word bet net).

**305- Conclusions**

309- Malaria infection, moreover, enhancing the utilization of ITNs among pregnant women (Replaced 'moreover' with 'and')
