# Supplementary material for: Utilization of insecticide-treated bed nets among pregnant women in Myanmar–analysis of the 2015–2016 Demographic and Health Survey
Source: PLoS One. 2022 Mar 10;17(3):e0265262. doi: 10.1371/journal.pone.0265262 (PMC8912190; doi:10.1371/journal.pone.0265262)
Supplement: Attachment — Submitted filename: REVIEWER RECOMMENDATIONS.dot [file pone.0265262.s004.dot]

**REVIEWER RECOMMENDATION and Comments of Manuscript Number** PONE-D-22-00065
Utilization of insecticide-treated bed nets to prevent malaria transmission among pregnant women in Myanmar – A secondary data analysis of the 2015-2016 nationwide Demographic Health Survey

Dr. Pyae Linn Aung

PLOS ONE

***Comments to the Author**

1. Does the manuscript provide a valid rationale for the proposed study, with clearly identified and justified research questions? Yes.

*2. Is the protocol technically sound and planned in a manner that will lead to a meaningful outcome and allow testing the stated hypotheses? Yes.

*3. Is the methodology feasible and described in sufficient detail to allow the work to be replicable? Yes.

*4. Have the authors described where all data underlying the findings will be made available when the study is complete? Yes.

*5. Is the manuscript presented in an intelligible fashion and written in standard English? Yes.

***6. Review Comments to the Author**

*7. PLOS authors have the option to publish the peer review history of their article (what does this mean?). If published, this will include your full peer review and any attached files. If you choose “no”, your identity will remain anonymous but your review may still be made public. Do you want your identity to be public for this peer review? Yes.

***Confidential to Editor**

*Confidential to Editor 1. Do you have any potential or perceived competing interests that may influence your review? No.

2. Did you receive any assistance in preparing this review. No

3. If accepted, do you think this submission should be highlighted on the PLOS ONE website? Accepted.
