# Supplementary material for: Utilization of insecticide-treated bed nets among pregnant women in Myanmar–analysis of the 2015–2016 Demographic and Health Survey
Source: PLoS One. 2022 Mar 10;17(3):e0265262. doi: 10.1371/journal.pone.0265262 (PMC8912190; doi:10.1371/journal.pone.0265262)
Supplement: Attachment — Submitted filename: Response to Reviewers.docx [file pone.0265262.s006.docx]

**Dear Editors and Reviewers.**

Thank you for the constructive comments, critiques, and suggestions. We have now attempted to address each point and provide a point-by-point response in **bold blue text** below. Page and Line numbers are referred to clean manuscript file.

**Journal Requirements:**

When submitting your revision, we need you to address these additional requirements.

1. Please ensure that your manuscript meets PLOS ONE's style requirements, including those for file naming. The PLOS ONE style templates can be found at

https://journals.plos.org/plosone/s/file?id=wjVg/PLOSOne_formatting_sample_main_body.pdf and

https://journals.plos.org/plosone/s/file?id=ba62/PLOSOne_formatting_sample_title_authors_affiliations.pdf

**RESPONSE:**

**Thank you for the suggestions. Now we have followed PLOS ONE’s style requirements including for files naming.**

2. We note that Figure 1 in your submission contain copyrighted images. All PLOS content is published under the Creative Commons Attribution License (CC BY 4.0), which means that the manuscript, images, and Supporting Information files will be freely available online, and any third party is permitted to access, download, copy, distribute, and use these materials in any way, even commercially, with proper attribution. For more information, see our copyright guidelines: http://journals.plos.org/plosone/s/licenses-and-copyright.

We require you to either (1) present written permission from the copyright holder to publish these figures specifically under the CC BY 4.0 license, or (2) remove the figures from your submission:

a. You may seek permission from the original copyright holder of Figure 1 to publish the content specifically under the CC BY 4.0 license.

We recommend that you contact the original copyright holder with the Content Permission Form (http://journals.plos.org/plosone/s/file?id=7c09/content-permission-form.pdf) and the following text:

“I request permission for the open-access journal PLOS ONE to publish XXX under the Creative Commons Attribution License (CCAL) CC BY 4.0 (http://creativecommons.org/licenses/by/4.0/). Please be aware that this license allows unrestricted use and distribution, even commercially, by third parties. Please reply and provide explicit written permission to publish XXX under a CC BY license and complete the attached form.”

Please upload the completed Content Permission Form or other proof of granted permissions as an "Other" file with your submission.

In the figure caption of the copyrighted figure, please include the following text: “Reprinted from [ref] under a CC BY license, with permission from [name of publisher], original copyright [original copyright year].”

b. If you are unable to obtain permission from the original copyright holder to publish these figures under the CC BY 4.0 license or if the copyright holder’s requirements are incompatible with the CC BY 4.0 license, please either i) remove the figure or ii) supply a replacement figure that complies with the CC BY 4.0 license. Please check copyright information on all replacement figures and update the figure caption with source information. If applicable, please specify in the figure caption text when a figure is similar but not identical to the original image and is therefore for illustrative purposes only.

**RESPONSE:**

**Figure 1 that is similar to a copyright figure has now been removed. Hope it will be fine.**

**REVIEWER:**

**Reviewer #1**: Thank you very much for this opportunity to revise the manuscript titled "Utilization of insecticide-treated bed nets to prevent malaria transmission among pregnant women in Myanmar – A secondary data analysis of the 2015-2016 nationwide Demographic Health Survey" that was submitted to PLOS ONE. The study is original research that utilized secondary data from 2015-2016 nationwide Demographic Health Survey-Myanmar. The study revealed very low rate of insecticide-treated bed nets usage among pregnant women. Insecticide-treated bed nets are the simplest way of preventing mosquito bites. Mosquito bites causes malaria. Malaria infection affects all persons in malaria endemic areas, however pregnant women and children under five are more vulnerable. In pregnancy, Malaria can cause anaemia, which lead to stillbirths and in some instance maternal morbidity and mortality. Due to the effectiveness of ITNs, most malaria endemic countries resort to free distributions to the population with special attention to the expectant mothers and postnatal mothers. However, in most countries the mere distribution of ITNs do not translate to utilisation. From above therefore, this study therefore is considered very important for policy directions. However, for this work to be published in Plos One, these are some comments for the authors in order to improve the manuscript:

Abstract
Methods under Abstract ought to be improved.
• Indicate the version of STATA used for the analysis.
• The study design is missing in this section.
• At what point, is a variable said to be significant in the inferential statistics?

**RESPONSE:**

**The abstract is improved. We stated as “The data analyzed in this cross-sectional study were extracted from available survey datasets of the 2015-16 Myanmar Demographic Health Survey. The secondary data were analyzed using a chart, descriptive statistics, and inferential statistics including simple and multiple logistic regression models. All analyses were performed using the STATA version 15. A p-value <0.05 was considered statistically significant.” (Page 2, Line 23-27)**

**REVIEWER:**

Introduction
-The authors begin talking about Insecticides Treated bed Nets. However, in many European countries, the use of this method of malaria prevention is little known, so it could be interesting to add a sentence explaining what it consists of and what health benefits it has. This will help readers form countries in which malaria is not endemic to appreciate the importance of this study.
The authors may refer to a study in Ghana for the definition and benefits of ITNs … Utilization of Insecticides Treated Mosquito Nets (ITNs) Among Pregnant Women in Kassena-Nankana Municipality… Kindly refer to the introduction and cite appropriately.

**RESPONSE:**

**Thank you so much for your suggestion. We have added a sentence describing a definition of ITN in Page 3, Line 56-57: “ITNs are a form of personal protection that has been proved to prevent malaria infection, and reduce the risk of severe sickness, and mortality due to malaria.” (Reference no. 4)**

**REVIEWER:**

-Kindly read the following published work in Myanmar and update line 78 to 81…. Other studies in Thailand and Myanmar stated that types of occupation, gender, age, socioeconomic status, net ownership, and residing places ….
Liu, H., Xu, J. W., Guo, X. R., Havumaki, J., Lin, Y. X., Yu, G. C., & Zhou, D. L. (2015). Coverage, use and maintenance of bed nets and related influence factors in Kachin Special Region II, northeastern Myanmar. Malaria journal, 14(1), 1-12.
Cheng, B., Htoo, S. N., Mhote, N. P. P., & Davison, C. M. (2021). Association between biological sex and insecticide-treated net use among household members in ethnic minority and internally displaced populations in eastern Myanmar. Plos one, 16(6), e0252896.

**RESPONSE:**

**Yes. We have gone through both publications and updated the sentence accordingly. Page 4, Line 82-86: Other studies in Thailand and Myanmar stated that types of occupation, sex, age, family wealth index, knowledge on malaria transmission, net ownership, household roofing, source of family income and geographical zones were associated factors for the use or nonuse of ITNs among the general population as well as migrant workers. (Reference no. 15 & 16)**

**REVIEWER:**

Methods

-Kindly specify if the study design is descriptive or analytical cross-sectional study

**RESPONSE:**

**The study design was analytical cross-section study. We have also added it in a paragraph under Methods section. (Page 5, Line 109-110).**

**REVIEWER:**

-Line 126 to 138 including the figure 1 should be moved to introduction.

**RESPONSE:**

**Thank you for the comment. We have moved the whole paragraph to Introduction part. (Page 4, Line 70-74) and (Page 4-5, Line 94-99)**

**REVIEWER:**

- Kindly make a sub heading for dependent (outcome) and independent (explanatory) variables and provide vivid description to each.

**RESPONSE:**

**We have revised data variables section as below. Page 6-7, Line 136-157 –**

**“The outcome variable of interest used in this study was currently pregnant women not sleeping under insecticide-treated nets (ITNs) the night before the survey. An ITN is defined as a factory-treated net that does not need to be treated again (long-lasting insecticide-treated net – LLIN), a pretreated net received during the last 12 months or a net that had been impregnated with insecticide within the last 9 months.**

**The independent predictor variables included first completed age, counted as the age in years each participant responded during the interview. The ranges were categorized into 15 to 24 years, 25 to 34 years, and 35 to 49 years. Second, highest education attained comprised the education attainments of the participants grouped as no education, primary, secondary, or higher levels. Third, place of residence and region consisted of regions categorized according to their characteristics: delta and lowland (Ayeyawady, Yangon and Bago Regions, Mon, and the Karen States), hilly (Kachin, Kayah, Chin and Shan States), coastal (Rakhine State and Tanintharyi Region) and plains (Magway, Mandalay, Sagaing Regions, and Nay Pyi Taw Union Territory). Fourth, wealth indexes, based on economic status, incomes, and property ownership, were differentiated into richer, richest, middle, poor and poorest. Fifth, number of household members included the total numbers of household members categorized in <3, 4 to 7, and >7. Sixth, number of under 5 children in the household totaled three groups for under 5 children, i.e., no one, one, or more than one. Seventh constituted duration of current pregnancy: the reported duration of pregnancy was coded in <5 months or 6 to 10 months. Finally, access to mass media exposure was defined as ‘yes’ when access could be gained to either one of television, newspaper, or radio at least once weekly.”**

**REVIEWER:**

- The criteria for inclusion into the logistics was not stated. This has the tendency affect the goodness of fit of the model.
See the following (concentrate on the data analysis only);
Mekonnen, B. D., & Wubneh, C. A. (2020). Prevalence and associated factors of contraceptive discontinuation among reproductive-age women in Ethiopia: using 2016 Nationwide Survey Data. Reproductive Health, 17(1), 1-10.
Abubakari, A., Taabia, F. Z., & Ali, Z. (2019). Maternal determinants of low birth weight and neonatal asphyxia in the Upper West region of Ghana. Midwifery, 73, 1-7.
Mutaru, A. M., Asumah, M. N., Ibrahim, M., Sumaila, I., Hallidu, M., Mbemah, J. M., ... & Zakaria, D. Y. (2021). Knowledge on Sexually Transmitted Infections (STIs) and sexual practices among Nursing Trainees in Yendi Municipality, Northern Region of Ghana. European Journal of Health Sciences, 6(4), 33-47.

**RESPONSE:**

**Thank you so much for pointing out the useful resources. We included age and education irrespective of their unadjusted p value (p value for association between the variable and ITN utilization). Age and education are known to be confounders and therefore included irrespective of their p value. We included other variables if their unadjusted p value was less than p<0.2. This was to ensure that the model was parsimonious. We including this information in the ‘Data analysis’ section. We hope this is fine.**

**REVIEWER:**

Results
-Table 1; kindly check the percentages of Wealth index, it is more than 100.0%. Kindly check the values at 1 decimal place again and make necessary changes.

**RESPONSE:**

**We have rechecked and ensured the percentage of each indicator to maintain at 100% in Table 1.**

**REVIEWER:**

-Line 194, the authors discovered that 96% of the pregnant women possessed bed nets. However, in figure 2 no such information is captured. kindly reconcile the descriptions and figure 2.

**RESPONSE:**

**Sorry for the confuse. We wrote that sentence based on the results from Table 1. Reference has now been cited accordingly. (Page 10, Line 200-202).**

**REVIEWER:**

-For consistency in reporting of findings …I suggest the authors reports findings as … 96% (95% CI: 93.2, 97.7)
-line 195-198, it is indicated that… “However, 16% (95% CI: 12.4, 20.2) of pregnant women slept without a bed net last night prior to the survey which was a bit higher than the general population (12%). Many of both groups preferred to sleep under the untreated nets. Only about one in five (18.4%, 95% CI: 14.7, 22.9) pregnant women slept under insecticide-treated bed nets”.

**RESPONSE:**

**We thoroughly checked this paragraph and ensured consistency in our writing. Page 10, Line 200-208: We revised as “Most pregnant women, 96% (95% CI: 93.2, 97.7), possessed mosquito bed nets for sleeping while only 33% possessed at least one insecticide-treated net (ITN) in their households (Table 1). However, 16% (95% CI: 12.4, 20.2) of pregnant women slept without a bed net the night before the survey. Many (65.7%) preferred to sleep under untreated nets. Only about one in five, 18.4% (95% CI: 14.7, 22.9) of total pregnant women slept under ITNs. Among pregnant women having access to at least one ITN in their households, almost 56% (86/154) slept under an ITN the night before the survey. Overall, about 84% and 88% of pregnant women and the general population slept in bed nets the night before the survey (Fig 1).”**

**REVIEWER:**

-From above, my understanding is that about 84% and 88% of pregnant women and general population respectively slept in bed nets a night to the survey. Is that correct?

-If above question is correct, how then did we get only 18.4% of pregnant women reported to be using insecticide-treated bed nets?
_How do we know that the other nets were not treated? In the introduction, I came across free distribution of bed nets aimed at meeting the 2030 targets by the country. It is the case that, majority of the 96% ownership of bed nets are not treated? Kindly explain and make it clear to the reader.

**RESPONSE:**

**Thank you for the comment. To be cleared, now we have added a new indicator in Table 1 that Having at least one insecticide treated net in the respondent household. It stated only 33% of study pregnant women possessed at least one ITN in their household while 96% possessed mosquito bed nets. Among pregnant women, 16% slept without net, 65.7% slept under untreated net, 18.4% slept under ITN. Among those who possessed at least an ITN in their household, about 56% slept under ITN. (Page 10, Line 200-208)**

**REVIEWER:**

-The authors further indicated that, only 18.4% slept in ITNs. this is problematic. Because the dependent (outcome) variable was not clearly defined, it is becoming very difficult to understand these findings.
-I would suggest that the methods be clarified to give true meaning to these findings.
-Bed nets and insecticide-treated bed nets should not be used interchangeably. Insecticide-treated bed nets are bed nets, but not all bed nets are insecticide-treated bed nets.
-I also suggest definition of terms at the methods to give true meaning to the findings.

**RESPONSE:**

**Thank you so much for the suggestions. Now we have revised and improved the variables section (Page 6-7, Line 136-157). We revised as “The outcome variable of interest used in this study was currently pregnant women who did not sleep under insecticide-treated nets (ITNs) last night prior to the survey. An ITN is defined as a factory-treated net that does not need to be treated again (long-lasting insecticide-treated net – LLIN) or a pretreated net received during the last 12 months or a net that has been impregnated with insecticide within the last 9 months.”**

**For the analysis (Page 7, Line 162-165), “The percentages were calculated for pregnant women who slept without nets, with untreated nets, with ITNs, respectively. To explore the true problem of ITN utilization status, utilization of ITN was estimated among total pregnant women and those with access to at least one ITN in their household. However (Page 7, Line 167-170), the variables related to the non-utilization of insecticidal nets among all pregnant women regardless of their ITN ownership status were explored by simple and multiple logistic regression models and presented using odds ratios with 95% confidence intervals.”**

**REVIEWER:**

-Line 207 -215 is not very necessary. I suggest to the authors to expunged expunge it.

**RESPONSE:**

**We have deleted it.**

**REVIEWER:**

-Only one variable is significant at the adjusted model because the authors did not control confounding variables. I would suggest that, only significant variables or variables with a p value less than 0.2 at bivariate analysis level be considered in the adjusted model analysis. This should be clearly stated at the methods. You may refer to the materials provided at the methods sections.

**RESPONSE:**

**Thank you so much for pointing out this. We described more details in the ‘Data analysis’ section. We stated as "The variables related to the non-utilization of insecticidal nets among pregnant women regardless of their ITN ownership status were explored using by simple and multiple logistic regression models and presented using odds ratios with 95% confidence intervals. We included age, education and variables with a crude Chi square p-value of <0.2 in the multiple logistic regression model.” .**

**REVIEWER:**

Discussions
-The discussion is okay and up to date, However, if authors would not mind, I would suggest they add some studies from Africa to support the work. I am suggesting very recent studies for your perusal and update. Africa is another malaria endemic area and almost all the interventions outline in the study are being done in most Africa countries.
Ngouakam, H., Fru-Cho, J., & Tientche, B. (2021). Awareness, Use, Care of Insecticide-treated Bed Nets among Pregnant Women in Buea (Buea) and Bonassama (Douala). Health Science Journal, 15(2), 1-8.
Asumah, M. N., Akugri, F. A., Akanlu, P., Taapena, A., & Boateng, F. (2021). Utilization of insecticides treated mosquito bed nets among pregnant women in Kassena-Nankana East municipality in the upper east region of Ghana. Public Health Toxicology, 1(2), 1-11. (Read to see why rural folks tend to use ITNs more than the urban folks, and the wealth index too at the discussion section)
Tassembedo, M., Coulibaly, S., & Ouedraogo, B. (2021). Factors associated with the use of insecticide-treated nets: analysis of the 2018 Burkina Faso Malaria Indicator Survey. Malaria Journal, 20(1), 1-9.

**RESPONSE:**

**Thank you so much for the useful resources. Of course, we have explored all of them and cited accordingly under the discussion part. (Reference no. 4, 19 & 29)**

**REVIEWER:**

-Limitation of this study should be mentioned at the end of the discussions.

**RESPONSE:**

**We have now added some sentences to describe the limitations of the study. Page 14-15, Line 303-311. “The study encountered a few limitations. First, it involved secondary data analysis of available data from countrywide demographics and health surveys. Therefore, the number of variables was limited. Other social-behavioral factors including misbeliefs, malaria knowledge and attitudes of pregnant women that might favor the non-utilization of ITNs need to be further explored. Second, the data obtained only by the questionnaire might under- or overestimate the actual situation. However, it might be escalated by observing ITN utilization during data collection visits. Third, the DHS survey was conducted from 2015 to 2016. Therefore, ITN ownership status might differ with current time due to rigorous distributions made after the country’s endorsement towards nationwide malaria elimination in 2015.”**

**REVIEWER:**

**Reviewer #2**: General comments
The overall structure of the manuscript is good and written based on the commonly agreed structure. The discussion and conclusion section needs major revision based on the objective and the result of the study.

Specific comments
1. Title: it can be modified “utilization of insecticide treated bed nets among pregnant women in Myanmar: analysis of 2015-2016 demographic and health survey”. Because no need of mentioning ---to prevent malaria transmission… as it is known.

**RESPONSE:**

**Now the title became – “Utilization of insecticide treated bed nets among pregnant women in Myanmar – analysis of the 2015-2016 Demographic and Health Survey.”**

**REVIEWER:**

2. Abstract
2.1 Background- it lacks information why the authors did the analysis.

**RESPONSE:**

**We have rewritten the Background section. “Due to the effectiveness of insecticide-treated nets (ITNs), most malaria-endemic countries resort to free distributions in the population with particular attention to pregnant women, a more vulnerable group. However, the mere distribution of ITNs does not usually translate to proper utilization. This study aimed to examine the utilization of ITNs to prevent malaria transmission and the underlying factors associated with the non-utilization of ITNs among pregnant women in Myanmar.” (Page 2, Line 17-22)**

**REVIEWER:**

2.2 Result- The description of the use of bed net is not clear. It stated that 16% slept without bed net and 18.4 slept under ITN. What about others…16% +18.4%=34.4%, 65.6% are they users or not. Make it clear for the reader. In the main document, it is clear.

**RESPONSE:**

**Now we have added another sentence for clearness. “Of 466 currently pregnant women, the majority (96%) possessed bed nets for sleeping. Among them, 16% slept without a bed net the night before the survey, while more than 65% slept with untreated nets. Only about 1 in 5 (18.4%) slept under ITNs.” (Page 2, Line 28-30)**

**REVIEWER:**

Mentioning the result of bivariate analysis in the abstract is not important.

**RESPONSE:**

**We have skipped the results of bivariate analysis.**

**REVIEWER:**

2.3 Conclusion- the recommendation sentence is not based on the significant variable. Mass media exposure do not show a significant association with utilization.

**RESPONSE:**

**We have improved the conclusion and omitted media exposure. Page 2, Line 35-38: “The relatively poor ITNs utilization rate was observed among pregnant women in Myanmar. To enhance the utilization of ITNs, targeted health education activities should be implemented. The priority group included vulnerable pregnant women residing in urban areas of the delta, lowland, plain and hilly regions without access to mass media exposure.”**

**REVIEWER:**

Keywords: it is good if arranged alphabetically

**RESPONSE:**

**We have rearranged the keywords in alphabetical order: Insecticide-treated nets, Myanmar, pregnant women, utilization. (Page 2, Line 39)**

**REVIEWER:**

3. Introduction
• Reference number one is outdated you can update and use the 2021 malaria report which indicates 241 million cases of malaria. (<https://www.who.int/news-room/fact-sheets/detail/malaria>)

**RESPONSE:**

**Now the sentence was updated with the numbers from the latest world malaria report. Page 3, Line 44-45: In 2020, the World Health Organization (WHO) declared almost 241 million malaria cases and 0.6 million deaths in the world.**

**REVIEWER:**

• Based on line 51 and 52 sentence, malaria is not a problem of pregnant women. How do the authors explain the public health importance of this research for Myanmar?

**RESPONSE:**

**We have rechecked the national data and corrected the number accordingly. The sentence should be read as - In 2019, a total of 56,640 malaria cases and 14 malaria-related deaths were reported in Myanmar, and 365 cases involved pregnant women. (Page 3, Line 50-51)**

**REVIEWER:**

• It is good if the authors indicate the interventions by different stakeholders to increase ownership and utilization of ITN among pregnant women.

**RESPONSE:**

**In the introduction part, we have updated the sentences for your consideration. Page 3, Line 64-69: “all malaria projects conducted mass distribution of insecticidal nets, either ITNs or Long-lasting Insecticide-treated Nets (LLINs) (hereafter named ITN), every other year according to the National Strategic Plan. The topping up replenishments covering malaria vulnerable groups, including forest-related workers, pregnant women, and children, were followed each year until the next cycle of mass distribution.”**

**REVIEWER:**

4. Method
• It is good if line number 126 to 138 presented under the introduction section, which shows the intervention by different stakeholders.

**RESPONSE:**

**Thank you for the comment. We have moved the whole paragraph to Introduction part. (Page 4, Line 70-74) and (Page 4-5, Line 94-99)**

**REVIEWER:**

5. Result
• Table 1- the bracket under percentage column for all percent is not necessary as it is written in a separate column. In the table the frequency added to 99.9% not 100%, check it.

**RESPONSE:**

**It’s true. We have removed all the brackets from Table 1 and 2.**

**REVIEWER:**

• Line 196 seems discussion, not description of the result. It can be stated in the discussion section.

**RESPONSE:**

**We have removed it accordingly.**

**REVIEWER:**

• Line 207-215 should be under bed net utilization, not the factors associated with bed net utilization.

**RESPONSE:**

**According to a suggestion from one other reviewer, we believe this part is not necessary and seems duplicated with the things inside the table. We have removed the paragraph.** **We hope this is fine.**

**REVIEWER:**

6. Discussion
• The discussion should be guided by the objective of the study. However, most of the parts of the discussion is based on the general understanding not based on the result of the study. Only paragraph 2 and 3 focused discussion based on the objective.

**RESPONSE:**

**Thank you for your suggestion. We have improved the discussion section and put more relevant literature and studies accordingly. Although some variables showed insignificant results, we believed it better to discuss the reason behind it. So that we could know the situation of the study area and formulate lessons to apply in other similar areas.**

**REVIEWER:**

• It lacks the limitation of the study. Many variables may be missed because it is a secondary data such as knowledge, attitude, risk perception etc.

**RESPONSE:**

**We have now added some sentences to describe the limitations of the study. Page 14, Line 303-311. “The study encountered a few limitations. First, it involved secondary data analysis of available data from countrywide demographics and health surveys. Therefore, the number of variables was limited. Other social-behavioral factors including misbeliefs, malaria knowledge and attitudes of pregnant women that might favor the non-utilization of ITNs shall be further explored. Second, the data obtained only by the questionnaire might under- or overestimate the actual situation. However, it might be escalated by observing ITN utilization during data collection visits. Third, the DHS survey was conducted from 2015 to 2016. Therefore, the ITN ownership status might differ with current time due to rigorous distributions made after the country’s endorsement towards nationwide malaria elimination in 2015.”**

**REVIEWER:**

7. Conclusion
• The recommendation part (line 310-318) is a general recommendation not based on the result of the study. Mass media and other intervention suggested is not the significant variables, which is associated with utilization of ITN in this study.

**RESPONSE:**

**The conclusion part was improved and omitted a fact about mass media. Page 14-15, Line 303-311: “The study encountered a few limitations. First, it involved secondary data analysis of available data from countrywide demographics and health surveys. Therefore, the number of variables was limited. Other social-behavioral factors including misbeliefs, malaria knowledge and attitudes of pregnant women that might favor the non-utilization of ITNs need to be further explored. Second, the data obtained only by the questionnaire might under- or overestimate the actual situation. However, it might be escalated by observing ITN utilization during data collection visits. Third, the DHS survey was conducted from 2015 to 2016. Therefore, ITN ownership status might differ with current time due to rigorous distributions made after the country’s endorsement towards nationwide malaria elimination in 2015.”**

**REVIEWER:**

**Reviewer #3**: The authors focused on the ownership and utilisation insecticide treated nets among pregnant women in Myanmar. Women constitute with children, a major risk group and known to be vulnerable to the disease. Despite great achievement in malaria control during the past decade, due to various interventions, the disease remains a huge public concern in developing countries. This is due partly to low utilisation of ITNs and poor attitude to preventive measures. Therefore, this paper presented aimed at to understand factors associated with the non-utilization of ITNs among pregnant women in Myanmar. The manuscript revealed a relatively poor ITNs utilization rate pregnant woman in Myanmar which could jeopardise the attainment of the malaria elimination goal by 2030. Residing in plains and hilly areas were preditors of non-utilisation of ITNs among pregnant women in Myanmar. With regards to weakness, minor English revision may be useful. The manuscript was well written following PLOS ONE recommended outline. The manuscript represents a high level of scientific work and appear to be an interested topic for scientists works in the areas of ITNs universal coverage.

**RESPONSE:**

**Thank you so much for the constructive comments. We thoroughly rechecked the Language of the manuscript and improved as necessary.**

**REVIEWER:**

**The Title**: Utilization of insecticide-treated bed nets to prevent malaria transmission among pregnant women in Myanmar – A secondary data analysis of the 2015-2016 nationwide Demographic Health Survey. **Proposed Title**: Utilization of insecticide-treated bed nets among pregnant women in Myanmar – a secondary data analysis of the 2015-2016 nationwide demographic health survey.

**RESPONSE:**

**Yes. Now the title has been changed to Utilization of insecticide treated bed nets among pregnant women in Myanmar – analysis of the 2015-2016 Demographic and Health Survey.**

**REVIEWER:**

**17- Abstract:**

**18- Background**: Myanmar is approaching to achieve the malaria elimination goal by 2030 (Please the authors should remove this sentence)

**RESPONSE:**

**We have removed it.**

**REVIEWER:**

**29-Results:** Among them, 16% slept without a bed net last night prior to the survey and only about one in five (18.4%) slept under ITNs. Among them, 16% slept without a bed net the night prior to the survey while only about one in five (18.4%) did.

**RESPONSE:**

**Yes. We have revised according to the suggestion. (Page 2, Line 29-30)**

**REVIEWER:**

32-33-34: Please when the results of the adjusted analysis are given, include the p-value. example: Women who were residing in the delta and lowland region [adjusted Odd Ratio (aOR)=7.97, 95% Confidence Interval (CI): 3.73, 17.05], p-value, plains (aOR=7.16, 95%CI: 3.11, 16.46, p-value)

**RESPONSE:**

**Yes. First, to know the true significance of variables, we put the 95%CI following each odd ratio. We have also added p-value whenever necessary under the Results section.**

**REVIEWER:**

**37- Conclusions**: The authors stated that "To enhance the utilization of ITNs, targeted health education using mass media should be implemented". Can the authors ascertained that in Myanmar do this intervention is yet to be implemented in Myanmar?

**RESPONSE:**

**Thank you for your suggestions. As per comments also from other reviewers, now we have revised the conclusion part by omitting a word about mass media. Page 2, Line 35-38: The relatively poor ITNs utilization rate was observed among pregnant women in Myanmar. To enhance the utilization of ITNs, targeted health education activities should be implemented. The priority group included vulnerable pregnant women residing in urban areas of the delta, lowland, plain and hilly regions without access to mass media exposure.**

**REVIEWER:**

**44-INTRODUCTION**

52-53. In Myanmar, 56,640 malaria cases and 14 malaria-related deaths were reported in 2019 and of them, none were reported as pregnant women (The authors should kindly rephrase this sentence).

**RESPONSE:**

**The sentence has been rephrased as - In 2019, a total of 56,640 malaria cases and 14 malaria-related deaths were reported in Myanmar, and 365 cases involved pregnant women. (Page 3, Line 50-51)**

**REVIEWER:**

74-75 Thus, it needs immediate attention to strengthen the use of ITNs especially in rural areas.

(Thus, there is a need for immediate attention to strengthen the use of ITNs especially in rural areas).

**RESPONSE:**

**It has been revised accordingly. Page 4, Line 78-80: Thus, a need exists for immediate attention to strengthening the use of ITNs especially in rural areas where malaria is endemic and in other malaria vulnerable groups like pregnant women.**

**REVIEWER:**

**97-Methods**: Well written and clearly stated

**179- RESULTS**

184-185. Many respondents 185 (59.3%) occupied 4-7 household members (Kindly rephrase the sentence).

**RESPONSE:**

**The sentence has been rephrased as many respondents (59.3%) possessed 4-7 members in their households. (Page 8, Line 190-191)**

**REVIEWER:**

194 Most of the pregnant women (96%, 95% CI: 93.2, 97.7) Please kindly include the p-value when reporting the Odd ratio.

**RESPONSE:**

**Yes, we have added p-value whenever necessary throughout the manuscript.**

**REVIEWER:**

195-196-However, 16% (95% CI: 12.4, 20.2) of pregnant women slept without a bed net last night prior to the survey which was a bit higher than the general population (Please the authors should kindly represent the p-value. This is a result section and the authors should not consider any comparison with the general population

**RESPONSE:**

**Thank you so much for the suggestion. Yes, we have added p-value whenever necessary. The sentence was rewritten as - However, 16% (95% CI: 12.4, 20.2) of pregnant women slept without a bed net the night before the survey. (Page 10, Line 202-204)**

**REVIEWER:**

197-Many of both groups preferred to sleep under the untreated nets (Please the authors should kindly rephrase this sentence).

**RESPONSE:**

**It was rewritten as - Many (65.7%) preferred to sleep under untreated nets. (Page 10, Line 203-204)**

**REVIEWER:**

208 nets (ITNs) last night before the survey. The night before the survey

**RESPONSE:**

**Yes. It was revised.**

**REVIEWER:**

211-212-213. Moreover, the ones who had the lowest wealth index (91.2%) and possessed 4 to 7 family members in their households (82.7%) did not utilize ITNs last night before the survey. (Moreover, those who had the lowest wealth index (91.2%) and possessed 4 to 7 family members in their households (82.7%) did not utilize ITNs the night before the survey).

**RESPONSE:**

**This sentence has been removed as per recommendation from one other reviewer as the information is duplicated what was described in the table. We hope it will be fine.**

**REVIEWER:**

215 Last, the majority (85.2%) were exposed to mass media at least once a week. (Please removed the word last at the beginning of the sentence).

**RESPONSE:**

**This sentence has been removed as per recommendation from one other reviewer as the information is duplicated what was described in the table.**

**REVIEWER:**

216- In bivariate analysis (In the bivariate analysis).

**RESPONSE:**

**Yes. It was revised. (Page 10, Line 216)**

**REVIEWER:**

**209. Discussion**

245-246. In this study, although most of the pregnant women possessed a kind of bed nets (The authors should clarify the kind of bed nets).

**RESPONSE:**

**Yes. The sentence was revised. In this study, although most pregnant women possessed either treated or untreated nets, some did not sleep under bed nets while only a few slept under ITNs. (Page 12, Line 245-247)**

**REVIEWER:**

245- 246. In this study, although most of the pregnant women possessed a kind of bed nets, some did not sleep under bed nets while only a few slept under ITNs. (Authors should reconsider this sentence, there is a repetition of the word bet net).

**RESPONSE:**

**Yes. The sentence was revised. In this study, although most pregnant women possessed either treated or untreated nets, some did not sleep under bed nets while only a few slept under ITNs. (Page 12, Line 245-247)**

**REVIEWER:**

**305- Conclusions**

309- Malaria infection, moreover, enhancing the utilization of ITNs among pregnant women (Replaced 'moreover' with 'and')

**RESPONSE:**

**Yes. It was revised. (Page 15, Line 317)**
