# Supplementary material for: Utilization of insecticide-treated bed nets among pregnant women in Myanmar–analysis of the 2015–2016 Demographic and Health Survey
Source: PLoS One. 2022 Mar 10;17(3):e0265262. doi: 10.1371/journal.pone.0265262 (PMC8912190; doi:10.1371/journal.pone.0265262)
Supplement: Attachment — Submitted filename: Response to Reviewers.docx [file pone.0265262.s007.docx]

**Dear Editors and Reviewers.**

Thank you for the constructive comments, critiques, and suggestions. We have now attempted to address each point and provide a point-by-point response in **bold blue text** below. Page and Line numbers are referred to clean manuscript file.

**Journal Requirements:**

Please review your reference list to ensure that it is complete and correct. If you have cited papers that have been retracted, please include the rationale for doing so in the manuscript text, or remove these references and replace them with relevant current references. Any changes to the reference list should be mentioned in the rebuttal letter that accompanies your revised manuscript. If you need to cite a retracted article, indicate the article’s retracted status in the References list and also include a citation and full reference for the retraction notice.

**Response**

**We have thoroughly checked the entire references list and changed as necessary. Particularly, the reference number 23 was duplicated with reference 10 so that we deleted it and revised the sequences of references numbers in the manuscript text as well as in reference list. In addition, the whole manuscript was thoroughly checked and improved the language with the help of a native speaker with eminent background of malaria.**

**Reviewer**

Reviewer #1: Thank you very much for this opportunity to revise the manuscript titled "Utilization of insecticide-treated bed nets to prevent malaria transmission among pregnant women in Myanmar – A secondary data analysis of the 2015-2016 nationwide Demographic Health Survey" that was submitted to PLOS ONE. The study is original research that utilized secondary data from 2015-2016 nationwide Demographic Health Survey-Myanmar.

Abstract
*The secondary data 25 were “analyzed” using a chart, … Please replace analyzed to “presented”

**Response**

**We have changed the word “analyzed” to “presented”. (Page 2, Line 24)**

**Reviewer**
* Among them, 16% slept without a bed net the night before the survey, while more 30 than 65% slept with untreated nets. Only about 1 in 5 (18.4%) slept under ITNs. (16+65+18.4=99.4%) where is the remaining 0.6%.

*Introduction and methods are sound in my view.

**Response**

**We have rechecked the percentages and revised the sentence as below.**

**Among them, 15.9% slept without a bed net the night before the survey, while 65.7% slept with untreated nets. Only about 1 in 5 (18.4%) slept under ITNs. (Page 2, Line 28-29). We believed total percentage is now equal to 100%.**

**Reviewer**
Results
*In Table 1, please reference to the last two variables. The study reported that 96% of the pregnant have or owned or possessed a bed net. Yet only 33.0% were reported to have had ITNs. Please, are you sure these subjects could differentiate between bed nets and ITNs? You may have to have something around this in your limitation if you intend to leave it as captured now.

**Response**

**Thank you for pointing out this fact. We have inserted sentences under limitations.**

**Second, the data obtained only by the questionnaire might under- or overestimate the actual situation. Respondents might also wrongly report between conventional bed nets and ITNs. However, these might have been escalated by data collection visits conducted during the DHS by observing ITN utilization. (Page 14-15, Line 304-308)**

**Reviewer**
*In line 206 to 208, results on the general population are presented. Please, the general population is not your sample units. I think we should not report on that.

**Response**

**We have now excluded the result representing for general population.**

**Page 10, Line 203-204; Overall, about 84% of pregnant women slept in bed nets the night before the survey.**

**Reviewer**
*In table 2, the second variables which is level of education has a p value of (0.389) at the bivariate level (COR). Given that you have set p<0.2 as the inclusion criteria into the adjusted model, it ought not to be part of the variables entered into the multivariate analysis (AOR). Kindly take it out.
*Kindly bold the significant variables.

**Response**

**Thank you. As suggested, we excluded ‘education’ in the final model and revised the numbers accordingly throughout the text as well as in the table.**

**Moreover, the significant variables are bold. (Table 2, Page 11)**

**Reviewer**
*Kindly add something about the importance of this study just after the limitations. For examples, “… Despite these limitations, this study provides essential information which could be relied upon to improve upon the effective utilization of ITNs especially among the pregnant women and perhaps children under five; since they are vulnerable to malaria…” Feel free to rephrase to suit your work.

**Response**

**Thank you for your suggestion. We have added a sentence as follow.**

**Despite these limitations, this study provides essential information which could be relied upon to improve the effective utilization of ITNs especially among pregnant women and perhaps children under five; since they are vulnerable to malaria. (Page 15, Line 310-313)**

**Reviewer**

Reviewer #2: General comments
The authors’ addressed most of my comments and made significant improvements of the manuscript. For further improvement of the manuscript, here under I suggest few modifications.

Specific comments
Abstract
Background- (Line 20-22) - the last paragraph that states the objective has to be rephrased. My suggestion “this study aimed to examine the utilization of ITN and associated factors among pregnant women in Myanmar”. Because utilization can be yes or no or utilized and non-utilized.

**Response**

**The objective has been rephrased. Page 2, Line 20-21, This study aimed to examine the utilization of ITNs and its associated factors among pregnant women in Myanmar.**

**Reviewer**
Conclusion- (Line 36-38) - in the recommendation part, still I am not convinced the suggestion of mass media. First, mass media exposure did not show any relation with utilization or non-utilization of ITN. Second, there is no evidence in this study whether the mentioned regions lack access to media. I think it is preferable to recommend to the government or any concerned organizations to give more emphasis in those regions to increase the level of ITN utilization. Probably suggesting further study to identify the factors that is not addressed by this study. There are many important factors missed in this research such as knowledge of ITN, attitude towards ITN, perceived susceptibility to malaria, etc.

**Response**

**The conclusion was revised as necessary. Information about mass media have now been excluded. Now it should be read as -**

**(Page 2, Line 34-38) - Relatively poor ITN utilization was observed among pregnant women in Myanmar. Health promotion activities for ITN utilization should be implemented especially for pregnant women residing in the delta, lowland, plain and hilly regions. Other social-behavioral factors including perceived susceptibility to malaria, knowledge of ITNs, and attitude towards ITN that might favor the non-utilization of ITNs need to be further explored.**

**Reviewer**
Discussion
Line 284-294- is all about the general population, not related with pregnant women, which is the study population for this research. If I am not mistaken, are pregnant women involved in Gold mining and rubber tapping? If they are involved, it is acceptable explanation. May be you can check the reason for not utilizing is due to priority given to their children, if there is a culture of priority given to children in your country, etc. than discussing the reason of the general population.

**Response**

**The paragraph has now been revised to express especially for pregnant women. Information regarding gold mining and rubber tapping are omitted.**

**(Page 14, Line 280-291); Many studies concluded that the individual's economic status was associated with levels of malaria preventive practice in the community. Mostly, ITNs are distributed free of charge directly to the hands of pregnant women. Meanwhile, the country’s GDP is at the lowest level. Universal Health Coverage is still in the skilling up phase. Rural families with financial hardship are trying hard to earn a decent income for their daily survival and healthcare seeking. Therefore, most are usually remote from routine malaria control interventions including awareness-raising campaigns. As per local culture, additionally, women always give priority to their children or other family members. When ITN ownership status is not high, pregnant women cannot utilize the ITN although they possess good malaria preventive knowledge. Need-based targeted interventions are recommended to take place among underprivileged individuals with low economic status in need of support. All families should possess at least one ITN per two persons in their households.**

**Reviewer**

Conclusion
See my comments in the conclusion section of the abstract.

**Response**

**The inclusion part is improved as necessary. Especially, we adjusted some usage and skipped the information about mass media. (Page 15, Line 316-325) –**

**Although the bed net ownership status was high, a relatively poor ITN utilization rate was observed among pregnant women in Myanmar. It may jeopardize the attainment of countrywide malaria elimination by 2030. Eliminating unwarranted maternal mortality due to malaria transmission and enhancing the utilization of ITNs among pregnant women is essential. Targeted health education activities among pregnant women should be implemented throughout the country. Next, healthcare professionals should implement routine health education sessions or awareness-raising campaigns for pregnant women during antenatal care visits to foster malaria preventive practices. The priority group includes vulnerable pregnant women residing in urban areas of the delta, lowland, plain and hilly regions.**
